# Impact of pharmacogenetics on aspirin resistance: a systematic review

**DOI:** 10.1055/s-0042-1758445

**Published:** 2023-03-14

**Authors:** Gustavo Figueiredo da Silva, Bruno Mattei Lopes, Vinicius Moser, Leslie Ecker Ferreira

**Affiliations:** 1 Universidade da Região de Joinville, Departamento de Medicina, Joinville SC, Brazil; 2 Universidade da Região de Joinville, Joinville Stroke Biobank, Joinville SC, Brazil

**Keywords:** Pharmacogenetics, Aspirin, Genetic Variation, Farmacogenética, Aspirina, Variação Genética

## Abstract

**Background** Pharmacogenetics promises better control of diseases such as cardiovascular disease (CVD). Acetylsalicylic acid, aspirin, prevents the formation of an activating agent of platelet aggregation and vasoconstriction, and it is used to prevent CVD. Nevertheless, patients may have treatment failure due to genetic variants that modify the metabolism of the drug causing aspirin resistance (AR).

**Objectives** To realize a systematic literature review to determine the impact of genetic variants on AR.

**Methods** Articles published in the MEDLINE/PubMed, Cochrane, Scopus, LILACS, and SCIELO databases were systematically screened. A total of 290 articles were identified and 269 articles were excluded because they did not comply with the previously established inclusion criteria. A total of 20 case-control studies and 1 cohort was included.

**Results** The genetic variants rs1126643 ( *ITGA2* ), rs3842787 ( *PTGS1* ), rs20417 ( *PTGS2* ), and rs5918 ( *ITGB3* ) were the most studied. As for relevance, of the 64 genetic variants evaluated by the articles, 14 had statistical significance ( *p* < 0.05; 95% confidence interval [CI]) in at least one article. Among them, the following have had unanimous results: rs1371097 ( *P2RY1* ), rs1045642 ( *MDR1* ), rs1051931 and rs7756935 ( *PLA2G7* ), rs2071746 ( *HO1* ), rs1131882 and rs4523 ( *TBXA2R* ), rs434473 ( *ALOX12* ), rs9315042 ( *ALOX5AP* ), and rs662 ( *PON1* ), while these differ in real interference in AR: rs5918 ( *ITGB3* ), rs2243093 ( *GP1BA* ), rs1330344 ( *PTGS1* ), and rs20417 ( *PTGS2* ). As study limitations, we highlight the nonuniform methodologies of the analyzed articles and population differences.

**Conclusion** It is noteworthy that pharmacogenetics is an expanding area. Therefore, further studies are needed to better understand the association between genetic variants and AR.

## Resumo

**Antecedentes** A farmacogenética promete melhorar o controle de doenças como as cardiovasculares. O ácido acetilsalicílico, a aspirina, previne a formação de um agente ativador da agregação plaquetária e vasoconstrição e é usado na prevenção de tais doenças. No entanto, os pacientes podem ter falha no tratamento devido a variantes genéticas que modificam o metabolismo da droga causando resistência à aspirina (RA).

**Objetivos** Realizar uma revisão sistemática da literatura para determinar o impacto das variantes genéticas na resistência à aspirina.

**Métodos** Artigos publicados nos bancos de dados MEDLINE/PubMed, Cochrane, Scopus, LILACS e SCIELO foram sistematicamente selecionados. Foram identificados 290 artigos e, destes, 269 artigos foram excluídos por não atenderem aos critérios de inclusão previamente estabelecidos. Um total de 20 estudos caso-controles e 1 coorte foi incluído.

**Resultados** As variantes genéticas rs1126643 ( *ITGA2* ), rs3842787 ( *PTGS1* ), rs20417 ( *PTGS2* ) e rs5918 ( *ITGB3* ) foram as mais estudadas. Quanto à relevância, das 64 variantes genéticas avaliadas pelos artigos, 14 tiveram significância estatística ( *p* < 0,05; intervalo de confiança [IC] de 95%) em pelo menos um artigo. Entre eles, os seguintes tiveram resultados unânimes: rs1371097 ( *P2RY1* ), rs1045642 ( *MDR1* ), rs1051931 e rs7756935 ( *PLA2G7* ), rs2071746 ( *HO1* ), rs1131882 e rs4523 ( *TBXA2R* ), rs434473 ( *ALOX12* ), rs9315042 ( *ALOX5AP* ) e rs662 ( *PON* 1), enquanto estes diferiram na interferência real na RA: rs5918 ( *ITGB3* ), rs2243093 ( *GP1BA* ), rs1330344 ( *PTGS1* ) e rs20417 ( *PTGS2* ). Como limitações do estudo, destacam-se as metodologias não uniformes dos artigos analisados e as diferenças populacionais.

**Conclusão** Vale ressaltar que a farmacogenética é uma área em expansão. Portanto, mais estudos são necessários para entender melhor a associação entre variantes genéticas e RA.

## INTRODUCTION

Cardiovascular disease (CVD) is the first cause of mortality worldwide, with all the healthcare systems facing this very challenging issue. The World Health Organization (WHO) estimates that 31% of deaths worldwide are due to CVD, with ∼ 17.7 million CVD-related deaths in 2015. Approximately 7.4 million of these deaths were due to heart disease and 6.7 million deaths were due to stroke. 1 Platelet activation plays an important role in the development of CVD. Acetylsalicylic acid (ASA), commonly known as aspirin, is an irreversible inhibitor of platelet cyclooxygenase (COX), which prevents the formation of thromboxane A2 by arachidonic acid and, therefore, prevents the formation of this activating agent of platelet aggregation and vasoconstriction. 2 Aspirin is a widely used antiplatelet for primary and secondary prevention of CVD, such as stroke and heart attacks. 3

Nevertheless, several patients may still experience treatment failure with ASA and an increased risk in recurrent stroke events. 4 There are several contributing factors for treatment failure including medication adherence, drug-drug interactions, aspirin-independent thromboxane A2 synthesis and also genetic variations. 2 Even low daily aspirin doses (in the range between 75 and 150 mg) are able to suppress biosynthesis of thromboxane, inhibiting the accumulation of platelets, and reducing the risk of CVD. 5 However, aspirin does not always prevent the formation of thromboxane A2 due to failure to inhibit platelet COX. 6 Because of that, all individuals do not respond to antiplatelet therapy in a similar way. In this sense, the genetic mutations have been related with aspirin resistance (AR) and may cause reduction or increase in drug absorption and metabolism, contributing to AR. 6 7

Aspirin resistance can be diagnosed by clinical criteria or by laboratory tests. Clinically, the patient has a new episode of CVD, despite the regular use of aspirin. While the failure of aspirin to inhibit a platelet function test can be seen by Platelet Function Analyser (PFA-100) or light transmission aggregometry (LTA), for example. 3

The field of pharmacogenetics, which aims to implement specific pharmacological therapies to genetic characteristics with the intention to provide greater efficiency, is a constant target of research. 8 Therefore, several studies have been published about candidate genes associated with the genetic predisposition of resistance to AAS, such as *COX-2* , *GPIIIA* , and *P2Y1* . 9 Resistance to antiplatelet therapy and the indiscriminate use of ASA can increase rates of recurrence and mortality from cardiovascular diseases, such as stroke. 10 Hence, the aim of the present study was to perform a systematic literature review to determine the impact of genetic variants on AR.

## METHODS

The present systematic review was established according to the recommendations of the Preferred Reporting Items for Systematic Reviews and Meta-Analyzes (PRISMA) statement published by Moher et al. (2019). Five following databases were systematically screened: MEDLINE/PubMed, 11 Cochrane, 12 Scopus, 13 LILACS, 14 and SCIELO. 15 The research was restricted to a period of 10 years (December 2009 to December 2019) and the following search terms were applied: *Aspirin* AND *Resistance* AND *Polymorphism* and *Aspirin* AND *Resistance* AND *Genetic variation* .

### Eligibility criteria

Only articles published in English were included in this search. Also, only articles describing the relation between AR, proven by laboratory tests or a new case of CVD, and polymorphisms or genetic variations were included in the present systematic review. The final articles included ( *n =* 21) in the present review were 20 case-controls and 1 cohort.

### Assessment of risk of bias

The authors, using the combined search terms and based on the inclusion criteria, conducted the primary literature search. In that first moment, titles and abstracts were screened. All reports that appeared in accordance with the inclusion criteria were full-text screened. All studies that did not comply with pre-established eligibility and inclusion requirements were excluded. In a second step, the researchers independently evaluated whether the full-texts previously selected followed the inclusion criteria. In case of disagreement between two authors, a third author was consulted, and a consensus was reached by a meeting between them.

Furthermore, to assess and minimize the presence of potential biases, the Risk of Bias in Systematic Reviews (ROBIS) method was used as a reference. 16

### Data extraction and synthesis

In the primary literature search, a total of 290 articles were found: 178 in SCOPUS, 104 in MEDLINE/Pubmed, 5 in Cochrane, 2 articles in LILACS, and 1 in SCIELO. Of those, 19 were duplicated. Hence, 271 articles were screened for reading of title and abstract, 216 of which were excluded for not meeting our inclusion criteria.

**Figure 1:**
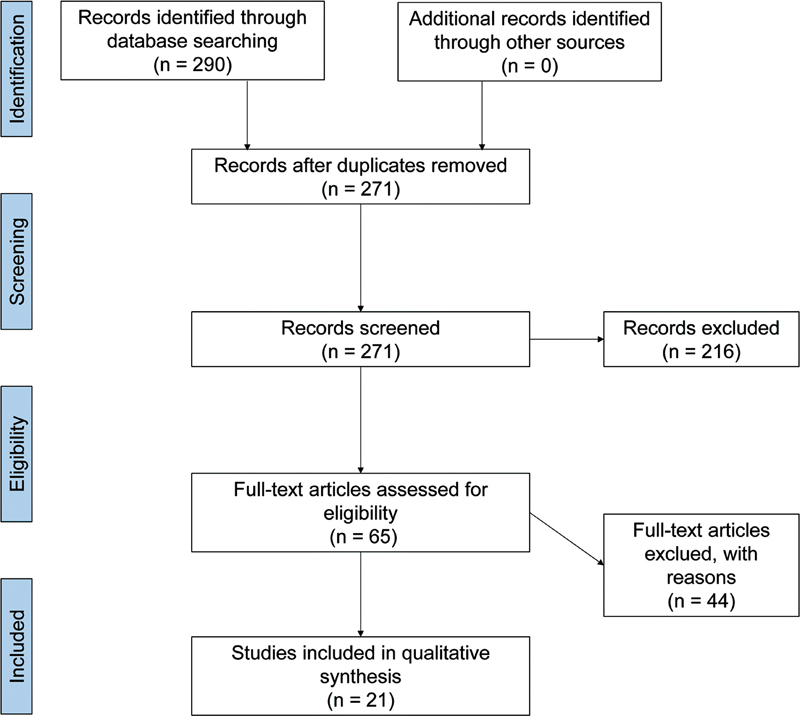
Flowchart of selected articles.

In the next step, the authors independently reviewed 65 full-text articles. Then, 44 articles were excluded for not meeting our inclusion criteria. So, in the end, 21 articles were included in the present systematic review ( Figure 1 ).

## RESULTS

In the 21 final articles selected, a total of 10,873 patients were analyzed, of which 3,014 were aspirin resistant and 6,882 were aspirin sensitive (some articles brought semiresistance values and were disregarded, and another 2 articles did not classify their patients as sensitive and not sensitive). Of the 21 articles studied, 11 included patients with a cerebrovascular event, totaling 4,835 patients. The other 10 articles mostly analyzed cardiac outcomes. We also emphasize that the clinical conditions of the evaluated patients were varied among the articles, with some articles evaluating patients with > 1 disease: ischemic stroke (10 articles), coronary artery disease (9), peripheral arterial disease (3), acute vascular event (1), age > 80 years old (1), adults (1), and hypertension (1). Most of the patients in the selected articles are from the Asian continent (9 from China, 4 from India, 2 from Turkey, and 1 from Jordan), and regarding the other works, 3 articles are from the American continent (all from the United States of America), 1 from the European continent (Belgium), and 1 from the African continent (Tunisia).

Among the resistance analysis methods, 4 articles used clinical outcome and 17 used platelet aggregation measurement. Among those who performed platelet aggregation measurement, the most common method was LTA (8 articles), followed by PFA-100 system (3), thromboelastography platelet mapping assay (TEG) (2), VerifyNow (2), PL-11 platelet analyzer (1), TXB2 elisa kit (1) and urinary 11-dehydro TXB2 (1), with some articles using > 1 method.

In Table 1 , we detail the following information from the 21 final articles included in the present review: Type of article, country, clinical condition, sample number, number of aspirin resistant patients, number of aspirin sensitive patients, gene, risk allele, protective allele, genetic variant, p-value, Odds Ratio (OR), CI, resistance assessment method, and daily aspirin dose.

**Table 1:** Compilation of the included articles

Author (year)	Type of article	Country	Clinical condition	Sample number*	Aspirin resistant	Aspirin sensitive	Gene	Protective allele	Risk allele	Genetic variation	*p-value*	OR	CI	Resistance assessment method	Aspirin dose/day
Patel S. et al (2019) 23	Case-control	India	Ischemic stroke	65	2	62	CYP2C19	G	A	rs4244285 (CYP2C19*2)	0.171	NI	NI	Platelet Aggregation Measurement - LTA	75mg
							ITGA2B/ITGB3	T	C	rs5918 (PLA1/A2)	0.960	NI	NI		
Yeo et al. (2018) 35	Cohort	USA	Peripheral artery disease	154	31	123	PTGS1	A	G	rs10306114 (A842G)	NI	NI	NI	Platelet Aggregation Measurement - VerifyNow Assay	300mg
							PTGS1	C	T	rs3842787 (C22T)	NI	NI	NI		
							PTGS1	C	A	rs5788 (C644A)	NI	NI	NI		
							PTGS1	C	A	rs5789 (C714A)	NI	NI	NI		
							ITGA2	C	T	rs1126643 (C807T)	NI	NI	NI		
							ITGA2	G	A	rs1062535 (873G/A)	NI	NI	NI		
							ITGA2	C	T	rs1126643 (C807T)	NI	NI	NI		
							ITGB3	T	C	rs5918 (PLA1/A2)	NI	NI	NI		
							GP6	C	T	rs1613662 (C13254T)	NI	NI	NI		
							P2RY12	C	T	rs1065776 (893C > T)	NI	NI	NI		
							F13A1	G	T	rs5985 (V34L)	NI	NI	NI		
							PON1	A	G	rs662 (A576G)	0.005	NI	NI		
Wang et al. (2017) 28	Case-control	China	Ischemic stroke	97	43	54	ITGA2	C	T	rs1126643 (C807T)	0.210	NI	NI	Platelet Aggregation Measurement - PL-11 platelet analyzer	100mg
							PTGS2	G	C	rs20417 (G765C)	0.69	NI	NI		
Strisciuglio et al. (2017) 36	Case-control	Belgium	Stable CAD patients undergoing elective PCI	597	NI	NI	NPPA	T	C	rs5065 (T2238C)	0.7	NI	NI	Platelet Aggregation Measurement - VerifyNow P2Y12	500mg
Yi et al. (2017) 19	Case-control	China	Ischemic stroke	850	175	630	PTGS1	C	T	rs1236913	0.99**	NI	NI	Platelet Aggregation Measurement - LTA	200mg (14 days) and follow-up with 100mg
							PTGS1	C	T	rs3842787	0.76**	NI	NI		
							PTGS2	A	G	rs689466	0.89**	NI	NI		
							PTGS2	G	C	rs20417	0.26**	NI	NI		
							TXAS1	G	A	rs194149	0.42**	NI	NI		
							TXAS1	T	C	rs2267679	0.53**	NI	NI		
							TXAS1	G	T	rs41708	0.72**	NI	NI		
							P2RY1	A	G	rs701265	0.48**	NI	NI		
							P2RY1	A	G	rs1439010	0.32**	NI	NI		
							P2RY1	C	T	rs1371097	0.01**	NI	NI		
							P2RY12	C	T	rs16863323	0.21**	NI	NI		
							P2RY12	G	A	rs9859538	0.16**	NI	NI		
							ITGB3	A	G	rs2317676	0.24**	NI	NI		
							ITGB3	A	G	rs11871251	0.51**	NI	NI		
Peng et al. (2016) 20	Case-control	China	Ischemic stroke	283	250	33	ABCB1	C	T	rs1045642	0.021	0.421	0.233–0.759	Platelet Aggregation Measurement - TXB2 ELISA kit	100mg
							TBXA2R	G	A	rs1131882	0.028	2.712	1.080–6.810		
							PLA2G7	A	G	rs1051931	0.023	8.233	1.590–42.638		
							PLA2G7	C	A	rs7756935	0.023	8.233	1.590–42.638		
							PEAR1	G	T	rs12566888	0.378	0.660	0.260–1.671		
							PEAR1	G	A	rs12566888	0.378	0.660	0.260–1.671		
Yi et al. (2016) 8	Case-control	China	Ischemic stroke	850	175	630	PTGS1	T	C	rs1236913	0.95**	NI	NI	Platelet Aggregation Measurement- LTA	200mg (14 days) and follow-up with 100mg
							PTGS1	C	T	rs3842787	0.78**	NI	NI		
							PTGS2	T	C	rs689466	0.82**	NI	NI		
							PTGS2	G	C	rs20417	0.42**	NI	NI		
Derle et al. (2016) 3	Case-control	Turkey	Acute vascular event	208	67	141	ITGB3	T	C	rs5918 (PLA1/A2)	0.277	NI	NI	Platelet Aggregation Measurement - PFA-100 system	100–300mg
Wang et al. (2014) 24	Case-control	China	> 80 years old	450	236	214	ITGB3	T	C	rs5918 (PLA1/A2)	0.002	NI	NI	Platelet Aggregation Measurement - LTA	100mg
Al-Azzam et al. (2013) 27	Case-control	Jordan	Adults	584	92	492	ITGA2	C	T	rs1126643 (C807T)	0.116	NI	NI	Platelet Aggregation Measurement - Multiplate Analyzer system	100mg
							GP1BA	T	C	rs2243093	0.003	NI	NI		
							PTGS2	G	C	rs20417	0.485	NI	NI		
Li et al. (2012) 29	Case-control	China	CAD, stroke, and peripheral artery disease	431	36	231	PTGS1	C	T	rs1888943	0.92	NI	NI	Platelet Aggregation Measurement - LTA	75–160mg
							PTGS1	A	G	rs1330344	0.1	NI	NI		
							PTGS1	C	T	rs3842787	0.92	NI	NI		
							PTGS1	G	A	rs5787	0.92	NI	NI		
							PTGS1	C	A	rs5789	1	NI	NI		
							PTGS1	G	A	rs5794	1	NI	NI		
							PTGS2	G	C	rs20417	1	NI	NI		
							PTGS2	C	G	rs5277	0.24	NI	NI		
							HO1	A	T	rs2071746	0.04	NI	NI		
Wang et al. (2013) 21	Case-control	China	Patientsunderwent primary OPCAB	210	62	148	TBXA2R	T	C	rs4523 (T924C)	0.001	4.479	1.811–11.077	Platelet Aggregation Measurement - LTA	100mg
							ITGB3	T	C	rs5918 (PLA1/A2)	NI	NI	NI		
							P2RY1	A	G	rs701265 (A1622G)	0.724	1.178	0.473–2.934		
							GP1BA	C	T	rs6065 (C1018T)	NI	NI	NI		
Sharma et al. (2013) 32	Case-control	India	Ischemic stroke	450	217	233	PTGS2	G	C	rs20417 (-765G/C)	CC: *p* = 0.016 GC: *p* = 0.02	CC:OR-3.157 GC: OR-1.745	CC: 1.241–8.033GC: 1.059–2.875	Clinical outcome	75–325mg
Sharma et al. (2013) 17	Case-control	India	Ischemic stroke	610	307	303	ALOX5AP	T	A	rs9315042 (SG13S114T/A)	<0.001	2.983	1.884–4.723	Clinical outcome	75–325mg
Fan et al. (2012)	Case-control	China	CAD, hypertension, peripheral artery disease and stroke	431	38	393	PTGS1	A	G	rs1330344	0.01	1.82	1.13–2.92	Platelet Aggregation Measurement - LTA and TEG Platelet Mapping Assay	75–100 mg
							PTGS1	C	T	rs1888943	0.59	NI	NI		
							PTGS1	C	T	rs3842787	0.66	NI	NI		
							PTGS1	G	A	rs5787	0.49	NI	NI		
							PTGS1	C	A	rs5789	1	NI	NI		
							PTGS1	G	A	rs5794	1	NI	NI		
Sharma et al. (2012) 33	Case-control	India	Ischemic stroke	560	338	222	ABCB1	C	T	rs1045642	0.012	1.85	1.142–3.017	Clinical outcome	75–325 mg/dia
Gao et al. (2011) 22	Case-control	China	Patients underwent primary OPCAB	262	23	239	GP1BA	C	T	rs6065 (C1018T)	1	NI	NI	Platelet Aggregation Measurement - LTA	100mg
							ITGB3	T	C	rs5918 (P1A1/A2)	1	NI	NI		
							P2RY1	A	G	rs701265 (A1622G)	0.991	NI	NI		
							TBXA2R	T	C	rs4523 (T924C)	0.01	NI	NI		
Chakroun et al. (2011) 31	Case-control	Tunisia	Stable CAD	125	NI	NI	PTGS1	C	T	rs3842787 (C50T)	Urinary TxB2: 0.1PFA-100: 0.43	NI	NI	Platelet Aggregation Measurement - PFA-100 system and Urinary 11-dehydro-TXB2	250mg
Voora et al. (2011) 26	Case-control	USA	Coronary stenosis ≥ 75%	3449	865	2584	GNB3	C	T	rs5443 (C825T)	> 0.05	Black: 1.15 White: 0.93	Black: 0.71–1.87 White: 0.82–1.07	Clinical Outcome	Two groups: < 81mg and > 81mg
							ITGA2	C	T	rs1126643 (C807T)		Black: 1.10 White: 0.99	Black: 0.82–1.46 White: 0.87–1.14		
							ITGB3	T	C	rs5918		Black: 1.03 White: 0.98	Black: 0.71–1.50 White: 0.85–1.13		
							GP6	A	G	rs1613662		Black: 0.89 White: 0.99	Black: 0.66–1.20 White: 0.86–1.15		
							GP1BA	T	C	rs2243093		Black: 0.84 White: 1.01	Black: 0.62–1.14 White: 0.86–1.18		
							PEAR1	A	C	rs2768759		Black: 1.05 White: 0.95	Black: 0.46–2.41 White: 0.83–1.09		
							VAV3	A	C	rs6583047		Black: 1.06 White: 1.02	Black: 0.80–1.42 White: 0.89–1.16		
							F2R	A	T	rs168753		Black: 0.96 White: 1.06	Black: 0.60–1.54 White: 0.91–1.23		
							THBS1	A	G	rs2228262		Black: 0.68 White: 1.03	Black: 0.34–1.36 White: 0.88–1.21		
							PTGS1	C	T	rs3842787		Black: 1.29 White: 1.06	Black: 0.94–1.77 White: 0.88–1.29		
							ADRA2A	G	C	rs1800544		Black: 0.98 White: 0.97	Black: 0.63–1.51 White: 0.85–1.10		
Pamukcu et al. (2010) 25	Case-control	Turkey	Stable CAD	126	30	96	F5	G	A	rs6025 (G1691A)	0.302	NI	NI	Platelet Aggregation Measurement - PFA-100 system	NI (The *p-value* for the difference between the resistant and sensitive groups was 0.681)
							F5	A	G	rs1800595 (A4070G - H1299R)	0.191				
							F2	G	A	rs1799963 (G20210A)	0.644				
							F13A1	G	T	rs5985 (V34L)	0.480				
							FGB	G	A	rs1800790 (G455A)	0.814				
							SERPINE1	A	G	rs1799889 (4G/5G)	0.656				
							ITGB3	T	C	rs5918 (HPA1a/b)	0.623				
							MTHFR	C	T	rs1801133 (C677T)	0.362				
							MTHFR	A	C	rs1801131 (A1298C)	0.421				
							ACE	Ins	Del	rs1799752 (ACE I/D)	0.713				
							APOB	G	A	rs5742904 (R3500Q)	1				
							APOE	T	C	rs429358 (C112R)	0.695				
							APOE	T	C	rs429358 (C158A)	0.695				
Carroll et al. (2010) 34	Case-control	USA	Candidates for interventional cardiology on aspirin therapy	81	27	54	ALOX12	A	G	rs434473	0.043	NI	NI	Platelet Aggregation Measurement - TEG Platelet mapping	Not uniform
							ALOX15B	G	A	rs4792147	0.440				
							ALOX12	G	A	rs1126667	0.580				
							ALOX15	G	A	rs3892408	NI				

Abbreviations: CAD, coronary artery disease; CI, confidence interval; LTA, light transmission aggregometry; NI, not informed; OPCAB, off-pump coronary artery bypass; PCI, percutaneous coronary intervention; TxB2, thromboxane B2.

Notes: *The number of semiresistants is not included.

**These *p* -values are the result of comparing the Aspirin Semiresistance + spirin Resistance group with the Aspirin Sensitive group. There is no individual comparison between aspirin resistance X aspirin sensitivity.

In addition, we have highlighted in a separate table the genetic variants with relevant results for AR ( Table 2 ). As for relevance, of the 64 genetic variants evaluated by the articles, 14 had statistical significance ( *p* < 0.05; 95%CI). Among them, the following polymorphisms have had concordant results so far: rs1371097 *(P2RY1* ), rs1045642 ( *MDR1* ), rs1051931 and rs7756935 ( *PLA2G7* ), rs2071746 ( *HO1* ), rs1131882 and rs4523 ( *TBXA2R* ), rs434473 ( *ALOX12* ), rs9315042 ( *ALOX5AP* ), and rs662 ( *PON1* ). In turn, these genetic variants differ in real interference in AR: rs5918 ( *ITGB3* ), rs2243093 ( *GP1BA* ), rs1330344 ( *PTGS* 1), and rs20417 ( *PTGS2* ).

**Table 2:** Genetic variants with relevant results for aspirin resistance

Biomarker (Pharmacogene)	Alleles	Refs.
PON1	rs662	35
P2RY1	rs1371097	19
ABCB1	rs1045642	20 33
TBXA2R	rs1131882, rs 4523	20 21
PLA2G7	rs1051931, rs7756935	20
ITGB3	rs5918	24
GP1BA	rs2243093	27
HO1	rs2071746	29
PTGS2	rs20417	17
ALOX5AP	rs9315042	17
PTGS1	rs1330344	29 30
ALOX12	rs434473	34

## DISCUSSION

To study the relationship between polymorphisms and AR, it is necessary to consider the resistance analysis mode, which can be performed in two ways: clinical or laboratory. In the first, the patient is considered resistant if there is a negative outcome (death or stroke for example). 17 In the second, several types of tests can be used, such as PFA-100, VerifyNow Aspirin, TEG, PL-11 platelet analyzer, serum and urinary TXB2, LTA, and multiplate analyzer. However, it is important to highlight that the measurement of platelet response to aspirin is highly variable, likely due to differing dependence of the arachidonic acid pathway between techniques. In our research, the most used laboratory method was the LTA, which is considered the gold standard for testing platelet function. 18

The relationship between polymorphisms and AR has been described by Yi et al. This study assessed the interaction with *PTGS1* (rs1236913 and rs3842787), *PTGS2* (rs689466 and rs20417), *TXAS1* (rs194149, rs2267679, and rs41708), *P2RY1* (rs701265, rs1439010, and rs1371097), *P2RY12* (rs16863323 and rs9859538), and *ITGB3* (rs2317676 and rs11871251) gene variants. In the laboratory analysis, only rs1371097 of the *P2RY1* gene, comparison CC x TT + CT, obtained statistical relevance ( *p* = 0.01), even after adjusting for other covariates ( *p* = 0.002; OR = 2.35; 95%CI: 1.87–6.86). In addition, using the generalized multifactor dimensionality reduction (GMDR) method, the following 3 sets of gene-gene interactions were significantly associated with AR: rs20417CC/rs1371097TT/rs2317676GG ( *p* = 0.004; OR = 2.72; 95%CI: 1.18–6.86); rs20417CC/rs1371097TT/rs2317676GG/AG ( *p* = 0.034; OR = 1.91; 95%CI: 1.07–3.84); rs20417CC/rs1371097CT/rs2317676AG ( *p* = 0.0025; OR = 2.28; 95%CI: 1.13–5.33). These high-risk interactive genotypes were also associated with a bigger chance of early neurological deterioration ( *p* < 0.001; Hazard Ratio [HR] = 2.47; 95%CI: 1.42–7.84). 19

Peng et al. (2016) also assessed genes related to thromboxane and others. The analyzed polymorphisms were *ABCB1* (rs1045642), *TBXA2R* (rs1131882), *PLA2G7* (rs1051931 and rs7756935) and *PEAR1* (rs12041331–rs1256888). There was statistical significance for 3 of them: rs1045642 ( *p* = 0.021; OR = 0.421; 95%CI: 0.233–0.759), rs1131882 ( *p* = 0.028; OR = 2.712; 95%CI: 1.080–6.810) and rs1051931–rs7756935 ( *p* = 0.023; OR = 8.233; 95%CI: 1.590–42.638), 20 while Wang Z. et al (2013) researched the association with *TBXA2R* (rs4523), *ITGB3* (rs5918), *P2RY1* (rs701265), and *GP1BA* (rs6065) polymorphisms. The only polymorphism significantly associated with AR was rs4523 ( *p* = 0.001; OR = 4.479; 95%CI = 1.811–11.077). 21

Another study that assessed the *TBXA2* and glycoprotein genes was done by Gao et al. *GP1BA* (rs6065), *ITGB3* (rs5918), *P2RY1* (rs701265), and *TBXA2R* (rs4523) genetic variations were researched, but only *TBXA2R* (rs4523) polymorphism was related ( *p* = 0.01). 22 In addition, Patel et al. also studied the *ITGA2B/ITGB3* polymorphisms. They analyzed the relationship with *CYP2C19* (rs4244285) and *ITGA2B* /I *TGB3* (rs5918) polymorphisms. However, no association was observed ( *p* = 0.171 and *p* = 0.960, respectively). 23

Moreover, still in the scope of glycoprotein genes, Derle et al. conducted a study with 208 patients with vascular risk factors. *ITGB3* (rs5918) polymorphism was screened, and the results showed that there was no significant difference in the presence of the C allele between the groups ( *p* = 0.277). In addition, in the relationship between the presence of the C allele and atherothrombotic stroke, no significant difference was found ( *p* = 0.184). 3

A study by Wang B et al. also analyzed the rs5918 *(PLA1/A2* ) polymorphism of the *ITGB3* gene. All 214 patients in the aspirin sensitive group had the *PLA1/A1* genotype and no patients with *PLA2/A2* were found. However, of the 236 patients in the AR group, 12 had *PLA1/A2* heterozygous genotype ( *p* = 0.002), finding a statistically significant differenc. 24

In the study by Pamukcu et al., 13 polymorphisms of 10 different genes were tested, including *ITGB3* . The genes *F5* (rs6025, rs1800595), *F2* (rs1799963), *F13A1* (rs5985), *FGB* (rs1800790), *SERPINE1* (rs1799889), *ITGB3* (rs5918), *MTHFR* (rs1801133, rs1801131), *ACE* (rs1799752 - Ins/Del), *APOB* (rs5742904), and *APOE* (rs429358 - C112R and C158A) were evaluated. However, there was no significant result for any polymorphism (p > 0.05). 25 Furthermore, in the case-control study by Voora et al, 11 polymorphisms of 11 different genes were assessed: *GNB3* (rs5443), *ITGA2* (rs1126643), *ITGB3* (rs5918), *GP6* (rs1613662), *GP1BA* (rs2243093), *PEAR1* (rs2768759), *VAV3* (rs6583047), *F2R* (rs168753), *THBS1* (rs2228262), *PTGS1* (rs3842787), and *ADRA2A* (rs1800544). When comparing the groups, there was no relationship ( *p* > 0.05). 26

Another research that studied some of the same genes was conducted by Al-Azzam et al.: *GP1BA* (rs1126643), *ITGA2* (rs2243093) and *PTGS2* (rs20417). Of these, only the *GP1BA* (rs2243093) gene was related ( *p* = 0.003), analyzing the presence of the C allele. 27 Additionally, Wang et al. (2017) conducted a study about the following polymorphisms: *ITGA2* polymorphism gene at rs1126643 and *PTGS2* polymorphism gene at rs20417. The authors found no association: *p* = 0.21 for rs126643 and *p* = 0.69 for rs20417. 28

Moreover, Yi et al. used Matrix-Assisted Laser Desorption/Ionization-Time Of Flight (MALDI-TOF) to link *PTGS1* (rs1236913 and rs3842787) and *PTGS2* (rs689466, and rs20417) with AR. The analysis showed that there was no statistical relevance for the relationship. Only when the gene-gene interaction (rs3842787 and rs20417) was evaluated, there was statistical significance: rs3842787/CT + rs20417/CC ( *p* = 0.016; OR = 2.36; 95%CI: 1.12–6.86), rs3842787/TT, CT + rs20417/CC ( *p* = 0.078; OR = 1.36; 95% CI: 0.82–2.01), and rs3842787/CT + rs20417/GC ( *p* = 0.034; OR = 1.78; 95%CI: 1.04–4.58). Highlighting the fact that, for the second combination, there is an invalid CI. 19

Another study that investigated polymorphisms of the *PTGS1* (rs1888943, rs1330344, rs3842787, rs5787, rs5789, rs5794) and *PTGS2* (rs20417, rs5277) genes was conducted by Li et al.; in addition to these two genes, a genetic variant of the *HO1* gene (rs2071746) was also tested. As a result, only two genetic variations were associated with AR. The rs2071746 polymorphism ( *HO1* gene) had statistical significance to genotype TT ( *p* = 0.04; OR = 1.40; 95%CI = 0.59–3.30) and T allele ( *p* = 0.04; OR = 1.70; 95%CI =1.02–2.79), while rs1330344 ( *PTGS1* gene) had significant results only when G was the risk allele and analyzed separately ( *p* = 0.02; OR = 1.77; 95%CI = 1.07–2.92). 29

Still on the *PTGS1* gene, Fan et al. investigated several polymorphisms of the *PTGS1* gene (rs1888943, rs1330344, rs3842787, rs5787, rs5789, and rs5794), but rs1330344 was the only significantly related to AR ( *p* = 0.01; OR = 1.82; 95%CI = 1.13–2.92; allele value) just in LTA + TEG analysis. 30 Moreover, another case-control study by Chakroun et al. investigated the relationship between rs3842787 polymorphism of the *PTGS1* gene and AR. Patients with the allele had no statistically significant difference using CEPI-CT ( *p* = 0.1) and uTxB2 ( *p* = 0.43). 31

Sharma et al. evaluated 3 polymorphisms of 3 different genes, *PTGS2* (rs20417), *ALOX5AP (* rs9315042) and *ABCB1 (* rs1045642), to assess their role in AR. The research was performed in 3 different studies and all studies obtained statistical relevance for the CC allele of rs20417 ( *p* = 0.016; OR = 3.157; 95%CI: 1.241–8.033), the GC allele of rs20417 ( *p* < 0.001; OR = 2.983; 95%CI: 1,884–4,723) and for the rs9315042 variant ( *p* < 0.001; OR = 2.983; 95%CI: 1.884–4.723). For the variant rs1045642, 2 comparisons were made, one comparing cases and controls, for the TT x CC alleles ( *p* < 0.001; OR = 2.27; 95%CI: 1.64–3.168), and for the TT x CT + CC alleles ( *p* < 0.001; OR = 1.72; 95%CI: 1.335–2.239) and other comparing AR and sensitive participants ( *p* = 0.012; OR = 1.85; 95%CI: 1.142–3.017). 17 32 33

Another study that tested the *ALOX* gene was done by Carroll et al. The study tested 4 genetic variants: rs434473 and rs1126667 of the *ALOX12* gene, rs4792147 of the *ALOX15B* gene and rs3892408 of the *ALOX15* gene. Only the rs434473 polymorphism obtained a significant *p* -value ( *p* = 0.043). 34

Furthermore, Yeo et al. analyzed some variants of *PTGS1* (rs10306114, rs3842787, rs5788, and rs5789), *ITGA2* (rs1126643, rs1062535, and rs1126643), *ITGB3* (rs5918), *GP6* (rs1613662), *P2RY12* (rs1065776), and *F13A1* (rs5985) genes, but only rs662 ( *A576G* ) of *PON1* gene was significantly relevant ( *p* = 0.005) to AR. 35

Lastly, a study by Strisciuglio et al. included 450 noncarriers of the T2238C polymorphism (rs5065, *NPPA* gene) and 147 carriers. The authors concluded that there was no statistical difference when comparing the groups, neither in overall CAD patients ( *p* = 0.7) nor in the diabetic group ( *p* = 0.6). 36

As limitations of the present study, we highlight the nonuniform methodologies of the analyzed articles, as well as population differences. These divergences made it difficult to compare the results of the articles. Among the studies, there was a great difference among the clinical conditions, as well as in the way of analysis of the resistance and in the dosage of aspirin. Unfortunately, meta-analysis was not performed due to such high clinical and methodological heterogeneity of the findings.

Despite the heterogeneity of the findings in terms of methodology and results, it is clear that some polymorphisms are more studied than others. Among them, rs1126643 ( *ITGA2* ), rs3842787 ( *PTGS1* ), rs20417 ( *PTGS2* ), and rs 5918 ( *ITGB3* ) were the most studied.

In conclusion, pharmacogenetics is an expanding area that promises a therapy aimed at the individualities of each patient, personalized medicine, for better control of diseases, including cardiovascular diseases, such as stroke.

Finally, further studies are needed to better understand the association between genetic variants and AR and, therefore, the practical application of the findings.
